# Suppressed diversity of survivin splicing in active rheumatoid arthritis

**DOI:** 10.1186/s13075-015-0689-z

**Published:** 2015-07-10

**Authors:** Minna Turkkila, Karin M.E. Andersson, Sylvie Amu, Mikael Brisslert, Malin C. Erlandsson, Sofia Silfverswärd, Maria I. Bokarewa

**Affiliations:** Department of Rheumatology and Inflammation Research, Sahlgrenska University Hospital, Gothenburg University, BOX 480, SE-40530 Gothenburg, Sweden; Department of Microbiology, Tumor and Cell Biology, Karolinska Institutet, Nobels väg 16 FE280, 171 77, Stockholm, Sweden

## Abstract

**Introduction:**

Alternative splicing distinguishes normal and pathologic cells. High levels of oncoprotein survivin recognise patients with severe rheumatoid arthritis (RA). Here, we assess clinical relevance of alternative splicing of survivin in leukocytes of peripheral blood (PBMC) and bone marrow (BM) in RA patients.

**Method:**

Transcription of survivin wild-type (survivin-WT), survivin-2B and survivin-ΔEx3 was measured in 67 randomly selected RA patients and in 23 patients before and after B cell depletion with rituximab. Analysis was done in relation to disease activity, anti-rheumatic treatment and serum levels of rheumatoid factor (RF) and survivin.

**Results:**

Survivin-WT was the dominant splice variant equally expressed in T and B cells, while survivin-2B and survivin-ΔEx3 were higher in B cells. High disease activity (DAS28>5.1) was associated with an excess of survivin-WT and low ratios between survivin-2B/WT (p=0.035) and survivin-ΔEx3/WT in PBMC. Depletion of B cells by rituximab caused a decrease in survivin-WT (p=0.005) in PBMC, increasing the ratio between survivin-2B/WT (p=0.009) and survivin-ΔEx3/WT (p=0.001) in BM. This increase in survivin-2B/WT was associated with reduction in CD19+ BM cells (r=0.929, p=0.007), RF (IgM, r=0.857, p=0.024; IgA, r=0.739, p=0.021), and DAS28 (0.636, p=0.054). The increase in survivin-ΔEx3 in BM was associated with a reduction of CD19+ BM cells (r=0.714, p=0.058) and DAS28 (r=0.648, p=0.049), while survivin-ΔEx3/WT was associated with RF (IgG, r=0.882, p=0.016).

**Conclusion:**

This study demonstrates that the suppressed diversity of survivin splicing in leukocytes may attribute to adverse self-recognition in RA. Depletion of autoantibody producing B cells improves the balance of survivin splicing.

## Introduction

Survivin is a multifunctional protein that belongs to the inhibitor of apoptosis (IAP) family and is encoded by the *BIRC5* gene, which is found at chromosome 17q25 in humans [1]. Survivin is a marker of malignant cell growth expressed in a vast range of cancers (reviewed by [2]). In normal tissues, survivin is essential for fetal development and for regeneration and repair of damaged tissues [3].

Survivin has been identified in cytoplasm, nucleus and mitochondria and has different functions within these cellular localisations [4]. Nuclear survivin plays a part in regulation of cell division, whereas mitochondrial and cytoplasmic survivin inhibits apoptosis and promotes cell proliferation [5, 6]. Survivin is upregulated during the G2/M phase in mitosis and forms a chromosomal passenger complex together with inner centromere protein, Aurora B and borealin, aiding formation of microtubules and their attachment of kinetochores during cytokinesis [7]. When released from the nucleus, survivin displays anti-apoptotic functions. Cytoplasmatic survivin forms a complex with the X-linked IAPs (XIAP), which enhances its stability against ubiquitin-dependent degradation [8]. The XIAP–survivin complex binds caspase-3, preventing its pro-apoptotic functions. In the mitochondrial compartment, survivin binds pro-apoptotic protein Smac/Diablo that inhibits its release and activation of caspase-9 [9].

**Fig. 1:**
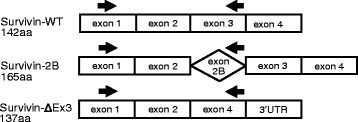
Survivin splice variants. Exon organisation in mRNA of human most frequent survivin splice variants, which comprise 98 % of mRNA expression from the *BIRC5* gene. Splice variants were measured using the same forward primer located in the N-terminus. The reverse primers were located within exon 3 (for survivin-WT), within the inserted sequence derived from intron 2 (for survivin-2B) and within the unique sequence of exon 4 (for survivin-ΔEx3). *Arrows* indicate positions of PCR primers. Sequences for each primer can be found in Methods. *UTR* untranslated region, *WT* wild type

The mRNA of human survivin has six different splice variants of which wild-type survivin (survivin-WT, 142 amino acids), survivin with an insert of additional exon 2 (survivin-2B, 165 amino acids) and survivin with depletion of exon 3 (survivin-ΔEx3, 137 amino acids) (Fig. 1) are the most frequent [10, 11] and comprise 98 % of mRNA expression from the *BIRC5* gene. All splice variants are identical in the N-terminus containing the BIR domain and differ in the carboxyl region. Survivin-WT and survivin-2B are actively moved out from the nucleus binding the carboxyl region to an exportin-1 [12, 13]. Survivin-ΔEx3 lacks the export signal, which is thought to keep it in the nucleus and in the mitochondria [5, 14].

Survivin-WT can form homodimers in solution and the balance between the dimer and monomer forms of survivin seems to regulate its ability to translocate and function in cellular compartments [15]. Additionally, survivin-WT may form heterodimers with survivin-2B and survivin-ΔEx3, which disrupts their normal function in cell death regulation and cell proliferation control [10, 14, 16]. Survivin-2B has a pro-apoptotic function [12, 17, 18] interfering with and blocking tubulin polymerisation and inducing mitochondria-dependent apoptosis [12, 17]. Survivin-ΔEx3 has dual functions. Similar to survivin-WT, it may inhibit apoptosis by preventing a XIAP-dependent activation of caspases in the cytoplasm and a release of Smac/Diablo from mitochondria [16, 19, 20]. In non-cancer cells, survivin-ΔEx3 mediates cell spreading, migration and stability [21]. If overexpressed, survivin-ΔEx3 also has a pro-apoptotic function and inhibits cell growth and proliferation in cell cultures [2, 22–24].

Overexpression of survivin in non-cancerous processes has been linked to inflammation, presumably contributing to the decreased apoptosis in the T cells of cerebrospinal fluid in multiple sclerosis [25], in skin lesions of patients with psoriasis [26] and in synovial tissue [27, 28] of patients with rheumatoid arthritis (RA). Reports on the role of survivin in the function of pluripotent stem cells [29, 30] and in the development of mature T cells [31] reserve a place for survivin-dependent mechanisms in immune responses. Our recent studies have shown an association between survivin and three key stones in the pathogenesis of RA: smoking [32], carriage of HLA-DRB1 antigen [33] and production of the RA-specific autoantibodies, rheumatoid factor (RF) and antibodies to citrullinated peptides (ACPA) [33–35]. High levels of survivin are associated with poor prognosis in RA predicting progressive joint damage and low responsiveness to anti-rheumatic treatment [35, 36].

In the present study we ask whether differential splicing of the survivin gene is of clinical relevance in RA. The measurement of three major splice variants of survivin showed that the proportional composition between survivin-WT and survivin-2B or survivin-ΔEx3 rather than the quantity of individual variants in leukocytes of peripheral blood (peripheral blood mononuclear cells (PBMCs)) and bone marrow (BM) is clinically important. An excess of survivin-WT and low survivin-2B/WT and survivin-ΔEx3/WT ratios in PBMCs distinguished patients with high disease activity, while anti-rheumatic treatment led to a gradual increase of the survivin-2B/WT ratio. Therapeutic B-cell depletion changed the profile of survivin splice variants in leukocytes of RA patients by reducing survivin-WT and favouring splicing of pro-apoptotic survivin-2B and survivin-ΔEx3. This change in survivin composition correlated to the reduction of B cells in BM, reduction in serum levels of RF and reduction in the disease activity. The association between survivin splicing and the autoreactive nature of B cells revealed a connection of survivin with the functional immune processes in RA.

## Methods

### Patients

A total of 67 randomly selected patients with established RA who attended the Rheumatology Clinic at Sahlgrenska University Hospital, Gothenburg, were included in the study. All patients fulfilled the 1987 Rheumatoid Arthritis Classification Criteria [37]. Clinical and demographic characteristics of the patients are presented in Table 1. At the time of blood sampling, 55 patients (82 %) were treated with methotrexate (MTX), and 23 of 55 patients combined MTX with biologics (tumour necrosis factor-alpha (TNFα) inhibitors, 10 patients; rituximab (RTX), 13 patients). One patient was treated with abatacept as monotherapy, and two with tocilizumab. The remaining nine patients had no treatment with disease-modifying anti-rheumatic drugs (DMARDs) at the time of the study. Another 23 RA patients were treated with 1000 mg B cell depleting monoclonal anti-CD20 antibodies (RTX; Hoffman-La Roche Ltd, Basel, Switzerland) provided intravenously on days 1 and 15 as described elsehere [38]. Disease activity was assessed at the day of blood sampling by evaluation of 28 joints for swelling and tenderness, and the Disease Activity Score (DAS28) was calculated.

**Table 1:** Clinical characteristic of patients with rheumatoid arthritis

	Cohort (*n* = 67)	Rituximab-treated group (*n* = 23)
Age (years)	56.6 (24–79)	59.4 (29–79)
Female/male	65/2	21/2
Disease duration (years)	10.2 (1–40)	13.1 (2–33)
RF positive (%)	83.6	96.0
ACPA positive (%)	71.7	83.0
DAS28	3.86 (1.38–6.77)	5.89 (4.6–6.8)
Methotrexate (*n*)	55 (82.1 %)	18 (78.3 %)
Dose (mg/week)	18.2 (7.5–25)	18.0 (10–25)
Anti-TNFα	10 (14.9 %)	0
Rituximab	13 (19.4 %)	9 (39.1 %)
Anti-IL-6R	2 (3.0 %)	0
CTLA4	1 (1.5 %)	0
Serum survivin (ng/ml)	18.5 (0.02–306.21)	2.07(0.10–14.9)

Values are mean and minimum and maximum are shown

*ACPA* antibodies against cyclic citrullinated peptides, *CTLA4* cytotoxic T lymphocyte associated protein, *DAS28* Disease Activity Score based on the evaluation of 28 joints, *IL-6R* interleukin 6 receptor, *RF* rheumatoid factor, *TNFα* tumour necrosis factor alpha

### Ethical approval

The study was approved by the Ethical Committee of the Sahlgrenska University Hospital. All patients gave written informed consent before giving blood and bone marrow samples.

### Blood and bone marrow sampling

Serum was prepared by centrifugation for 15 minutes at 800 × *g*, aliquoted and stored at −20 °C until analysed. PAXgene Blood RNA tubes (PreAnalytix, Hombrechtikon, Switzerland) were used for mRNA preparation according to the manufacturer’s recommendations. BM samples were obtained from 23 patients before RTX treatment by aspiration from the crista iliaca as described previously [38]. Blood and BM sampling was repeated 1 month or 3 months after the RTX treatment.

### Cell isolation and stimulation

Mononuclear cells from the peripheral blood (PBMCs) and BM were isolated by density gradient separation on Lymphoprep™ (Axis-Shield PoC As, Oslo, Norway). For in-vitro experiments, CD19^+^ B cells and CD4^+^ T cells were separated from the isolated PBMCs using Dynabeads® (Invitrogen, Carlsbad, CA, USA). The separated cell populations were resuspended in Dynal buffer (2 % bovine serum albumin (BSA) and 1 mM ethylenediamine tetraacetic acid (EDTA) in phosphate-buffered saline (PBS)) at a concentration of 2 × 10^6^ cells/ml. CD19^+^ cultures were stimulated with Pam3Cys (10 μg/ml; EMC Microcollections, Tubingen, Germany) and CD4^+^ cultures were stimulated with Concanavalin A (5 μg/ml; Sigma, St. Louis, MO, USA). Cells were collected after 72 hours, washed and lysed in RLT lysis buffer for RNA analysis.

### Serological measurements

Serum levels of survivin were measured using a matched antibody pair by a sandwich enzyme-linked immunosorbent assay (ELISA) (DYC647; R&D Systems, Minneapolis, MN, USA) as described previously [39]. Samples were diluted 1:10 and the detection limit was 0.1 ng/ml. Values above 0.45 ng/ml were defined as positive. Total levels of RF and ACPA antibodies were measured at the Laboratory of Clinical Immunology at Sahlgrenska University Hospital. RF of IgG, IgM and IgA subclasses were analysed from serum, diluted 1/1000, using an ELISA assay [38].

### RNA isolation and SYBR Green-based real-time PCR

Total RNA was extracted using the RNeasy Mini Kit (Qiagen, Valencia, CA, USA) and Paxgene Blood miRNA kit (Qiagen) according to the manufacturer’s recommendations. The RNA concentration and quality were measured using a Nanodrop spectrophotometer (ND1000 Spectrophotometer) and Experion™ RNA StdSens Analysis chip (Bio-Rad Laboratories, Hercules, CA, USA). cDNA was synthesised using the Applied Biosystems (Foster City, CA, USA) High-Capacity cDNA Reverse Transcription Kit according to the manufacturer’s recommendations. Transcription of survivin was assessed using SYBR Green qPCR Mastermix (SABiosciences, Qiagen) and ViiA™ 7 Real-Time PCR (Applied Biosystems). All samples used the identical forward primer GACCACCGCATCTCTACATTC and splice variant specific reverse primers (Fig. 1): survivin-WT, TGCTTTTTATGTTCCTCTATGGG; survivin-2B, AAGTGCTGGTATTACAGGCGT; and survivin-ΔEx3, ATTGTTGGTTTCCTTTGCATG [40] (Sigma). Melting curves for each PCR were performed between 60 and 95 °C to ensure specificity of the amplified product. All samples were run in duplicate with glyceraldehyde 3-phosphate dehydrogenase (GAPDH) as a reference gene and with a negative control. Expression levels of target genes were normalised to GAPDH to obtain the difference in cycle threshold (dCt). Six samples from healthy subjects were used as the reference group. The relative quantity (RQ) was calculated using the ddCt method.

### Statistical evaluation

Correlation between variables was analysed by Spearman test. Differences between two groups were tested by Mann–Whitney test and chi-square analysis. All tests were two-tailed and conducted with a 95 % confidence interval (CI) and *p* <0.05 was considered significant. Statistical analyses were performed using the Prism programme (v.6.x; GraphPad software, San Diego, CA, USA).

## Results

### Diversity of survivin splicing in leukocytes of the peripheral blood and BM

**Fig. 2:**
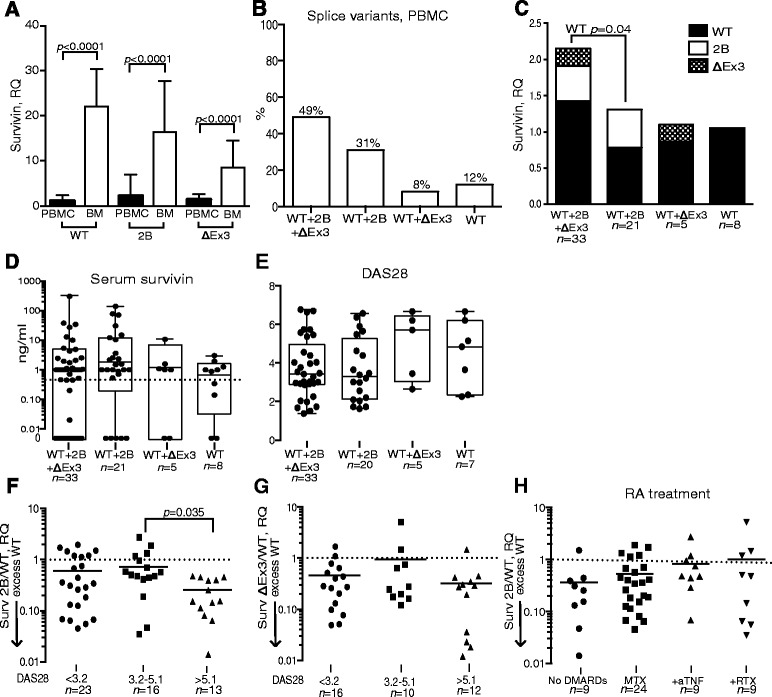
Levels of survivin splice variants in peripheral blood mononuclear cells (PBMCs) and bone marrow (BM) of patients with rheumatoid arthritis. **a** mRNA levels of survivin-WT, survivin-2B and survivin-ΔEx3 were measured in 67 PBMC samples and 23 BM samples and expressed as the relative quantity (RQ) of the reference group PBMC within each splice variant. **b** Combination of the splice variants in 67 PBMC samples. **c** Total mRNA level of survivin splice variants in PBMC samples, expressed as the RQ of survivin-WT in the group containing all splice variants. **d** Serum levels of survivin within the allocated groups. *Boxes* represent the 25th–75th percentile of the group; *horizontal lines* indicate median values and *error bars* show the range. **e** Disease Activity Score (DAS28) within the allocated groups. *Boxes* represent the 25th–75th percentile of the group; *horizontal lines* indicate median values and *error bars* show the range. **f** Survivin-2B/WT ratio and **g** survivin-ΔEx3/WT ratio in PBMCs for patients in remission (DAS <3.2), in active RA (DAS28 3.2–5.1) and in high active RA (DAS28 >5.1). *Lines* indicate mean values within each group. **h** Survivin-2B/WT ratio in PBMCs of patients with different treatment modalities. *Horizontal lines* indicate median value for the group. *DMARD* disease-modifying anti-rheumatic drug, *MTX* methotrexate, *+anti-TNF* combination of MTX and tumour necrosis factor inhibitors, *+RTX* combination of MTX and rituximab, *RA* rheumatoid arthritis, *WT* wild type

Transcription of survivin-WT, survivin-2B and survivin-ΔEx3 was analysed in 67 PBMC samples and in 23 BM samples of randomly selected RA patients. Clinical characteristics of the patients are presented in Table 1. The RQ of each survivin splice variant was much higher in the BM compared with PBMCs and showed approximately 20 times excess for survivin-WT, 15 times for survivin-2B, and seven times for survivin-ΔEx3 (Fig. 2a). Survivin-WT was the predominant splice variant present in all PBMC and BM samples. Survivin-2B and survivin-ΔEx3 were undetectable in 19–43 % of PBMC samples and 4–9 % of BM samples, which resulted in a diversity of survivin splicing in RA leukocytes (Fig. 2b). The level of survivin-WT was significantly higher (*p* = 0.04) in the samples combining expression of all three splice variants (Fig. 2c). The univariate analysis revealed no significant association between the levels of individual survivin splice variants and clinical parameters of RA (all *r* <0.3; data not shown). Serum levels of survivin (Fig. 2d), DAS28 (Fig. 2e) and disease duration (data not shown) were similar in the groups with different combinations of survivin splicing.

### Composition of survivin splicing and clinical signs of RA

Since survivin-WT may form dimers with survivin-2B and survivin-ΔEx3 opposing its functions, we studied the survivin-2B/WT (*n* = 54) and survivin-ΔEx3/WT (*n* = 38) ratios in PBMCs where both splice variants were present. We found that survivin-2B/WT was low and reflected a significant excess of survivin-WT in the patients with high disease activity (DAS28 >5.1) (Fig. 2f). Similarly, low survivin-ΔEx3/WT ratio with a relative excess of survivin-WT was found in PBMC samples irrespectively of DAS28 (Fig. 2g).

To study whether anti-rheumatic treatment affects the survivin-2B/WT ratio, we compared survivin splicing in patients with different treatment modalities. The survivin-2B/WT ratio increased gradually with the intensification of immunosuppression. This ratio was lowest in the patients having no DMARD treatment, and tended to increase in the patients treated with a combination of methotrexate with TNF inhibitors (Fig. 2h). The patients treated with a combination of MTX and RTX had further increase of the survivin-2B/WT ratio.

### Difference of survivin splicing in T and B lymphocytes

**Fig. 3:**
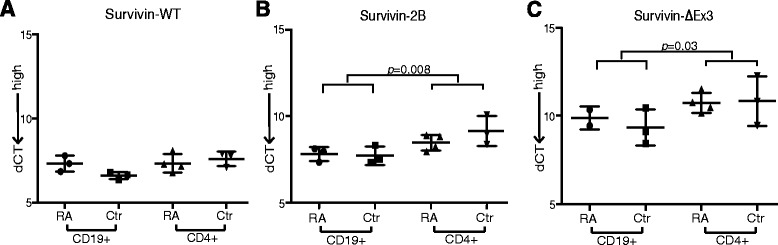
Survivin splice variants in CD19^+^ and CD4^+^ cells. **a** mRNA levels of survivin-WT, **b** survivin-2B and **c** survivin-ΔEx3 were measured in CD19^+^ and CD4^+^ cells isolated from the peripheral blood of RA patients (*n* = 4) and healthy controls (*n* = 3). Cells were activated for 72 hours to permit survivin production. *Error bars* show the range and *horizontal lines* indicate median values. Comparison between the groups was by Mann–Whitney statistics. *dCT* difference in cycle threshold, *RA* rheumatoid arthritis, *WT* wild type

To study composition of survivin splice variants produced by T and B lymphocytes, we separated CD4^+^ T cells and CD19^+^ B cells from PBMCs of four randomly selected RA patients (two women, two men) and of three healthy women. The separated cells were activated *in vitro* to permit survivin production, and mRNA levels of survivin-WT, survivin-2B and survivin-ΔEx3 were measured. The levels of survivin-WT were similar in CD4^+^ and CD19^+^ cells (Fig. 3a), while survivin-2B and survivin-ΔEx3 were significantly higher in CD19^+^ B cells (Fig. 3b and c, respectively). No significant difference in survivin-2B/WT and survivin-ΔEx3/WT ratio was found between RA patients and controls.

### B-cell depletion modifies survivin splicing in BM and PBMCs

**Fig. 4:**
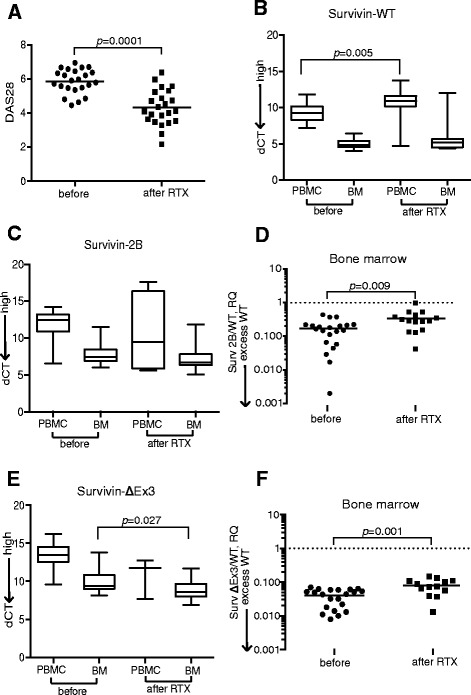
Survivin splicing in blood and bone marrow leukocytes of RA patients treated with rituximab (RTX). **a** Disease Activity Score (DAS28) before and after RTX treatment. mRNA level of **b** survivin-WT and **c** survivin-2B in peripheral blood mononuclear cell (PBMC) and bone marrow (BM) leukocytes of 23 patients before and 1–3 months after RTX treatment. **d** Survivin-2B/WT ratio in BM before and 1–3 months after treatment. **e** Survivin-ΔEx3 in PBMCs and BM before and 1–3 months after RTX treatment. **f** Survivin-ΔEx3/WT ratio in BM before and after RTX treatment. *Boxes* represent the 25th–75th percentile of each group, *horizontal lines* indicate median value and *error bars* show the range. Comparison between groups performed with Mann–Whitney statistics. *dCT* difference in cycle threshold between survivin and glyceraldehyde 3-phosphate dehydrogenase, *RQ* relative quantity

To assess whether B-cell depletion changed the splicing of the survivin gene we analysed the paired PBMC and BM samples of 23 RA patients before and after RTX treatment (Table 2). The RTX treatment resulted in the complete depletion of B cells from the PBMCs and a significant reduction of the B-cell population in the BM [40]. Consequently, a decrease of RF and of the disease activity (Fig. 4a) was observed. Low survivin-2B in the BM before RTX treatment was associated with higher numbers of BM CD19^+^ B cells (*r* = 0.857, *p* = 0.024).

**Table 2:** Effect of rituximab treatment in patients with rheumatoid arthritis

	Before RTX	After RTX^a^
DAS28 score	5.89 ± 0.6	4.34 ± 0.7
Rheumatoid factor (IU/ml)		
IgG	41.5 ± 29.3	22.7 ± 19.5*
IgM	67.2 ± 31.1	50.2 ± 38.3
IgA	41.0 ± 38.3	25.0 ± 19.5
WBC count (×10^9^/l)		
PBMCs	6.5 ± 3.1	7.8 ± 3.2
BM	2.1 ± 1.4	2.6 ± 1.6
CD19^+^ (%)^b^		
PBMCs	7.7 ± 3.8	0
BM	9.4 ± 4.3	1.3 ± 1.2^†^
CD3^+^ (%)^b^		
PBMCs	45.9 ± 15.8	42.5 ± 12.9
BM	28.0 ± 9.8	29.4 ± 16.5

Values presented as mean ± standard deviation, *n* = 23

^a^ Samples 1–3 months after RTX treatment

^b^ From the lymphocyte population

* *p* = 0.008

^†^ *p* <0.0001

*BM* bone marrow, *DAS28* Disease Activity Score based on evaluation of 28 joints, *PBMC* peripheral blood mononuclear cells, *RTX* rituximab, *WBC* white blood cell

**Fig. 5:**
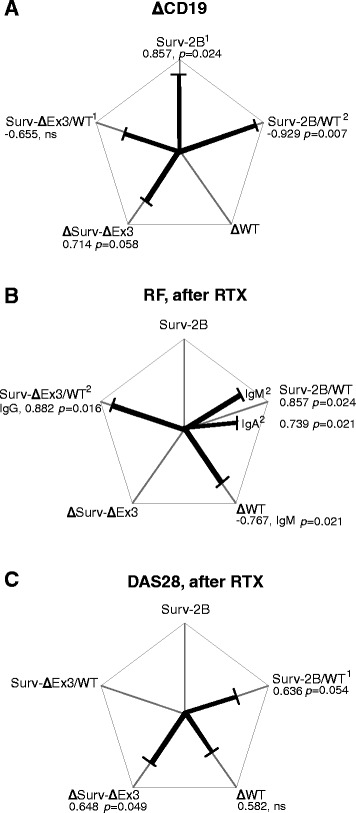
Correlations between clinical parameters of RA and survivin splice variants in BM after rituximab (RTX) treatment. Correlations between survivin splice variants and **a** change of CD19^+^ B cells, **b** levels of rheumatoid factor (RF) and **c** Disease Activity Score (DAS28) in 23 patients after RTX treatment. ^1^Correlation to survivin splice variants before RTX treatment. ^2^Correlation to survivin splice variants after RTX treatment. Correlations were calculated with Spearman’s analysis. dCT values were used for the individual splice variants, and RQ values were used for the survivin-2B/WT and survivin-ΔEx3/WT ratios. *WT* wild type

The B cell depletion in PBMCs was followed by a decrease of survivin-WT (Fig. 4b). The individual expression of survivin-2B in PBMCs and BM showed no statistical change (Fig. 4c). The change was, however, sufficient to attain a significant increase of the survivin-2B/WT ratio in BM (Fig. 4d). The reduction in survivin-WT and the change of the survivin-2B/WT ratio correlated to the reduction of the BM CD19^+^ B cells (Fig. 5a) and of RF levels (Fig. 5b). Remarkably, the association was strong for low-affinity RF-IgM antibodies and for the high-affinity RF-IgA. The reduction in survivin-WT and the increase of the survivin-2B/WT ratio was in direct proportion to the decrease of clinical activity of arthritis (Fig. 5c).

Following RTX treatment, survivin-ΔEx3 was increased in BM (Fig. 4e), which caused a significant increase of the survivin-ΔEx3/WT ratio (Fig. 4f). Analogously with survivin-2B, the change in survivin-ΔEx3 after RTX treatment correlated to a decrease in the BM CD19^+^ B cells (Fig. 5a) and in the serum survivin levels (*r* = 0.928, *p* = 0.008). The survivin-ΔEx3/WT ratio showed a correlation to the reduction of the high affinity RF-IgG antibodies (Fig. 5b).

## Discussion

This study shows that transcription of survivin in the leukocytes of PBMCs and BM has a tight connection to the pathogenesis of RA and to the functional autoimmune processes in these compartments. We present evidence that changes in the pattern of survivin splicing may belong to the clinically important events in RA.

Survivin-WT is the dominant survivin splice variant in PBMCs and BM of RA patients, followed by survivin-2B and survivin-ΔEx3, which confirms the observations in malignant cells and in pluripotent stem cells [29, 30, 41–43]. In the observational and in the B cell depletion parts of this study, the excess of survivin-WT is connected to high clinical activity of RA, suggesting that survivin-WT favours inflammatory and aggressive profile of leukocytes. Previous studies reported high expression of survivin in lymphocytes infiltrating tissue lesions in several non-cancer pathologies [25–28, 44]. However, splicing composition of this expression was not assessed.

This study showed that B cells from peripheral blood of RA patients and from healthy controls were recognised by high production of survivin-2B and survivin-ΔEx3. The low survivin-2B before RTX treatment and the increased production of survivin-2B and survivin-ΔEx3 thereafter was in direct relation to B cell numbers in the BM, and occurred in parallel with a decrease in survivin-WT. This observation leads us to suggest that IgD^+^ B cells depleted by RTX [38] are either the major source of survivin-WT or the suppressors of differential survivin splicing in other cells giving priority to survivin-WT. The elimination of IgD^+^ cells in BM of RA patients restrains the mechanisms of survivin splicing control.

The formation of autoreactive B cells measured by the levels of autoantibodies, RF and ACPA is a key to the aberrant immune responses in RA [45]. Depletion of B cells by RTX decreases serum levels of RF and ACPA [46–48], and leads to pronounced clinical improvement in RA patients [49]. Additionally, the presence of autoantibody-producing B cells has been indicated essential for the efficacy of RTX treatment [50, 51]. Here, we demonstrate that the reduction of serum RF-IgM had close association with a decrease of survivin-WT and with the changes in survivin-2B/WT ratio in the BM, suggesting that survivin-WT belongs to the autoreactive nature of B cells. RTX treatment affects homeostasis of B cells by elimination of the naïve and short-lived memory B cell from the peripheral blood and in the BM [38, 52, 53].

**Fig. 6:**
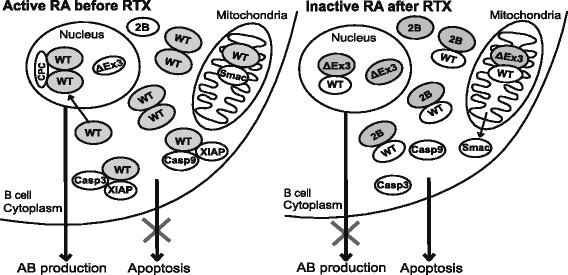
Graphic summary. Survivin-WT is by far the most prevalent variant of survivin in human leukocytes of the peripheral blood and of the bone marrow. Survivin-WT is known to be an efficient IAP, forming complexes with Smac/Diablo to prevent its release from mitochondria, and with X-linked inhibitor of apoptosis (XIAP) to inactivate caspase-3 (Casp3) and caspase-9 (Casp9). Homodimers of survivin-WT in the nucleus participate in the formation of chromosomal passenger complex supporting mitosis. Alternative splice variants survivin-2B and survivin-ΔEx3 have been suggested to form heterodimers with survivin-WT preventing its anti-apoptotic and pro-mitotic functions. Active rheumatoid arthritis (RA) is characterised by low survivin-2B/WT and survivin-ΔEx3/WT ratio indicating suppressed diversity of survivin splicing. The anti-rheumatic treatment, and particularly B-cell depletion with rituximab (RTX), increased the levels of survivin-2B and survivin-ΔEx3, potentially supporting formation of heterodimers with survivin-WT. The present study showed that this changed composition of survivin splice variants associates with a decrease in the number of CD19^+^ B cells, serum levels of autoantibodies (AB) and of the disease activity in patients with rheumatoid arthritis. *WT* wild type

We also found that the proportional composition between the anti-apoptotic survivin-WT and pro-apoptotic survivin-2B and survivin-ΔEx3 rather than the quantity of the individual splice variants was of clinical relevance in RA. Immunosuppressive treatment was associated with gradual increase of the survivin-2B/WT ratio (Fig. 6). Since both survivin-2B and survivin-ΔEx3 have been shown to oppose survivin-WT [54], a restriction of the anti-apoptotic function of survivin-WT in the cytoplasm and the mitosis-supporting function in the nucleus is expected. In the BM, this change in survivin composition of B cells could affect the formation and life-length of the antigen-experienced B cells, where the immature subsets of B cells may utilise the higher production of survivin-2B and survivin-ΔEx3 variants for their maturation limiting RF production. In the setting of RA, the excess of survivin-WT may be viewed as a mechanism to control repopulation and survival of autoantibody-producing B cells [38, 52]. Whether a reduction of survivin-WT or the changed balance in the composition of survivin splicing is responsible for the decrease in RA activity, however, is yet to be determined.

The mechanisms controlling survivin splicing in healthy cells and in pathology are poorly understood [24]. Aberrant regulation of survivin-2B and survivin-ΔEx3 has been recognised as a negative prognostic factor during breast, colorectal, renal and gastric carcinomas [2, 55]. The importance of differential tissue expression of survivin splice variants has been proposed, where diminished survivin-2B is associated with poor proliferation control and progressing tissue expansion. This is concordant with the results obtained in experimental arthritis demonstrating that inhibition of survivin translation by small hairpin RNA-expressing constructs results in the alleviation of joint inflammation and bone destruction [56]. Survivin inhibition achieved by these constructs was not splice variant specific [39], but reduction in the predominant splice variant, survivin-WT, could potentially have restored a balance between the pro-apoptotic and anti-apoptotic splice variants of survivin.

## Conclusions

Alternative splicing is generally viewed as a mechanism distinguishing healthy and pathologic cells. This study extends this perspective and demonstrates that suppressed diversity of survivin splicing occurred in active RA as a part of autoimmune inflammation. Depletion of autoreactive B cells contributes to changes of the survivin splicing profile and decrease of autoantibody production (Fig. 6).

## Abbreviations

ACPA: Anti-citrullinated protein antibody
BM: Bone marrow
BSA: Bovine serum albumin
CD19: Cluster of differentiation 19
CI: Confidence interval
CT: Cycle threshold
DAS28: Disease Activity Score
ΔEx3: delta exon 3
Diablo: Direct IAP binding protein with low pl
DMARD: disease-modifying anti-rheumatic drug
EDTA: Ethylenediamine tetraacetic acid
ELISA: Enzyme-linked immunosorbent assay
GAPDH: Glyceraldehyde 3-phosphate dehydrogenase
IAP: Inhibitor of apoptosis
MTX: Methotrexate
PBMC: Peripheral blood mononuclear cell
PBS: Phosphate-buffered saline
RA: Rheumatoid arthritis
RF: Rheumatoid factor
RQ: Relative quantity
RTX: Rituximab
SMAC: Second mitochondria-derived activator of caspases
TNFα: tumour necrosis factor alpha
WT: Wild type
XIAP: X-linked IAP
